# Correction: Development and validation of a cardiovascular risk prediction model for Sri Lankans using machine learning

**DOI:** 10.1371/journal.pone.0321407

**Published:** 2025-03-28

**Authors:** Chamila Mettananda, Isuru Sanjeewa, Tinul Benthota Arachchi, Avishka Wijesooriya, Chiranjaya Chandrasena, Tolani Weerasinghe, Maheeka Solangaarachchige, Achila Ranasinghe, Isuru Elpitiya, Rashmi Sammandapperuma, Sujeewani Kurukulasooriya, Udaya Ranawaka, Arunasalam Pathmeswaran, Anuradhini Kasturiratne, Norihiro Kato, Rajitha Wickramasinghe, Prasanna Haddela, Janaka de Silva

The 15^th^ author’s first name is incorrect. The correct name is: Norihiro Kato.
